# Prostate-Specific Membrane Antigen as Target for Neuroimaging of Central Nervous System Tumors

**DOI:** 10.1155/2022/5358545

**Published:** 2022-04-15

**Authors:** Brittany M. Stopa, James Crowley, Csaba Juhász, Cara M. Rogers, Mark R. Witcher, Jackson W. Kiser

**Affiliations:** ^1^Virginia Tech Carilion School of Medicine, Roanoke, VA, USA; ^2^Fralin Biomedical Research Institute, Roanoke, VA, USA; ^3^Carilion Clinic Radiology, Roanoke, VA, USA; ^4^Departments of Pediatrics, Neurology, Neurosurgery, Wayne State University School of Medicine, Detroit, MI, USA; ^5^PET Center and Translational Imaging Laboratory, Children's Hospital of Michigan, The Karmanos Cancer Institute, Detroit, MI, USA; ^6^Carilion Clinic Neurosurgery, Roanoke, VA, USA

## Abstract

**Introduction:**

Positron emission tomography (PET) imaging with prostate-specific membrane antigen- (PSMA-) binding tracers has been found incidentally to demonstrate uptake in CNS tumors. Following the encouraging findings of several such case reports, there is a growing interest in the potential application of PSMA-targeted PET imaging for diagnostics, theranostics, and monitoring of CNS tumors. This is a systematic literature review on PSMA-binding tracers in CNS tumors.

**Methods:**

A PubMed search was conducted, including preclinical and clinical reports. One hundred and twelve records were identified, and after screening, 56 were included in the final report.

**Results:**

Tissue studies demonstrated PSMA expression in tumor vascular endothelial cells, without expression in normal brain tissue, though the extent and intensity of staining varied by anti-PSMA antibody and methodology. Most included studies reported on gliomas, which showed strong PSMA ligand uptake and more favorable tumor to background ratios than other PET tracers. There are also case reports demonstrating PSMA ligand uptake in prostate cancer brain metastases, nonprostate cancer brain metastases, and meningiomas. We also review the properties of the various PSMA-binding radiotracers available. Therapeutic and theranostic applications of PSMA-binding tracers have been studied, including labeled alpha- and beta-ray emitting isotopes, as well as PSMA targeting in directing MRI-guided focused ultrasound.

**Conclusions:**

There is a potential application for PSMA-targeted PET in neuro-oncology as a combination of diagnostic and therapeutic use, as a theranostic modality for managing CNS tumors. Further research is needed regarding the mechanism(s) of PSMA expression in CNS tumors and its differential performance by tumor type.

## 1. Introduction

Standard imaging of central nervous system (CNS) tumors with magnetic resonance imaging (MRI) has demonstrated limitations [1], and positron emission tomography (PET) has emerged as an additional prominent noninvasive imaging modality for CNS tumors. Among the PET tracers under investigation for imaging of CNS tumors is prostate-specific membrane antigen (PSMA) binding tracer, whose utility in the CNS was originally noted on PET scans of patients with prostate cancer [2–4]. PSMA is a type II transmembrane glycoprotein that was originally identified due to its high expression in malignant prostate vasculature. Subsequent research has revealed that it is also expressed in a variety of other tissue types in different organ systems [2, 4, 5]. PSMA is also known as glutamate carboxypeptidase II (GCPII) in the brain, as well as folate hydrolase I in the intestines [6], where its main enzymatic function is to cleave N-acetyl-L-aspartyl-L-glutamate (NAAG) to N-acetylaspartate and glutamate [7]. In the normal rodent and human brain, astrocytes demonstrate some GCPII expression, which provides the majority of NAAG-hydrolyzing activity [6, 8], implicating this enzyme in some neuropsychiatric conditions in which glutamate is considered neurotoxic. As a result, inhibition of GCPII was thought to be neuroprotective, and GCPII inhibitor administration increased brain NAAG levels, improving cognitive performance in mice with experimental autoimmune encephalomyelitis [9]. Interestingly, efforts to develop small molecule inhibitors of GCPII to treat brain disorders of hyperglutamatergic pathogenesis led to the discovery of their application outside of the nervous system, most prominently as imaging and therapeutic agents in prostate cancers [10].

PET radioligands targeting PSMA are misleading in their nomenclature as “PSMA tracers,” because they are in reality inhibitors with high affinity for the PSMA-binding motif [11]. Such PSMA-targeting tracers have in recent years garnered greater interest because of their potentially widespread oncologic diagnostic applications, as well as therapeutic applications including those recently developed for prostate cancer. Importantly, PSMA is shown to have specific expression on tumor vasculature, which is unique from other vascular imaging tracers which nonspecifically bind to the vasculature in both normal tissue and tumor tissue, and this distinction may therefore facilitate direct targeting of tumor angiogenesis [12]. Case reports initially revealed the incidental finding of PSMA accumulation in CNS metastases, leading to subsequent exploration of PSMA expression and imaging in a variety of CNS malignancies. Here, we systematically review the literature investigating PSMA-targeted tracers for CNS lesions, including all identified *in vitro* and *in vivo* studies, in order to summarize current knowledge regarding the potential for PSMA-targeted PET in neuro-oncology imaging.

## 2. Methods

This review utilized a search strategy to identify previous preclinical and clinical research studies of PSMA-targeted PET in neuro-oncologic applications. The search was performed in the PubMed database (https://pubmed.ncbi.nlm.nih.gov) in May 2021 using the following search terms: ((brain tumor) OR (spine tumor) OR (CNS tumor) OR (neurooncology) OR (neuro-oncology) OR (glioblastoma) OR (glioma) OR (astrocytoma) OR (oligodendroglioma) OR (meningioma) OR (brain metastasis) OR (spine metastasis) OR (brain metastases) OR (spine metastases) OR (chordoma) OR (craniopharyngioma) OR (gangliocytoma) OR (glomus) OR (pineocytoma) OR (pituitary adenoma) OR (schwannoma) OR (acoustic neuroma) OR (ependymoma) OR (medulloblastoma) OR (hemangioblastoma) OR (rhabdoid)) AND ((positron emission tomography) OR (PET)) AND ((prostate specific membrane antigen) OR (PSMA)). All available years were included.

**Figure 1:**
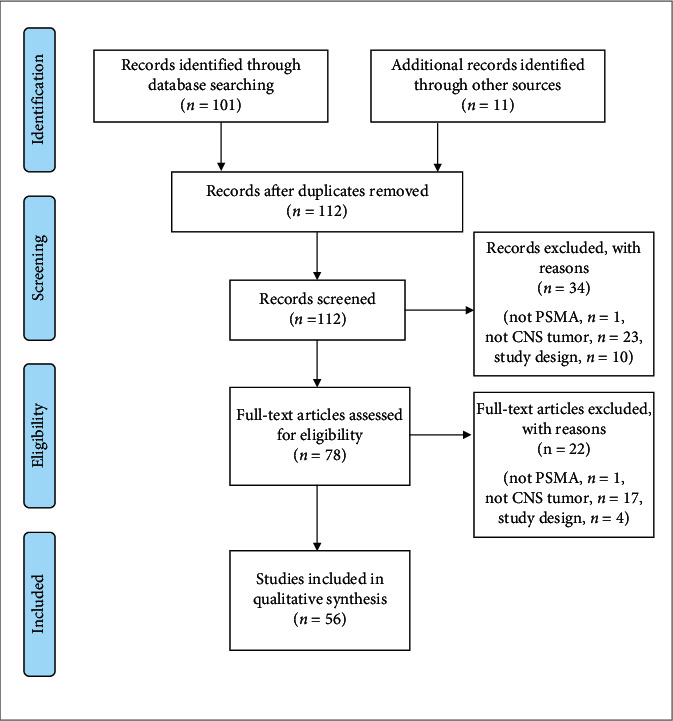
PRISMA study selection flowchart.

A total of 101 records were returned. The bibliographies of these papers were reviewed to elicit additional papers, yielding 11 additional articles. These 112 reports were then screened for original research publications related to PSMA and neuro-oncology, including preclinical studies, retrospective and prospective clinical studies, and case reports, but excluding review papers, systematic reviews, and meta-analyses. This yielded 56 studies for inclusion in the final review. See the study selection flowchart in Figure 1.

Data collection and extraction were performed independently by one author (BS) with oversight by the other authors. Data variables collected included year, study design, study subjects, tumor type, tracer name, and main study results. Data were analyzed and summarized qualitatively.

## 3. Results

### 3.1. Expression of PSMA in Tissue and Preclinical Models

There are 13 published reports of PSMA expression in CNS tumor tissue and preclinical models, including a collective total of 331 patients and 38 animals (Table 1). The first such report was by Chang et al. (1999), in which the expression profile of several PSMA antibodies in a wide variety of tumor tissue types, among them a single glioblastoma (GBM), were examined [13]. They reported that GBM neovasculature cells stained PSMA-positive, while neither tumor cells nor normal brain demonstrated expression.

Wernicke et al. (2011) published an immunohistochemistry study of 32 GBMs, using an anti-SMA mAb 3E6 (Dako) stain, quantifying the extent and intensity of vascular endothelial staining [14]. They reported that all GBMs stained positive for PSMA, with variable extent and intensity. The extent of staining was mostly 51-100%, while the intensity of staining was mostly moderate or maximum. The PSMA staining colocalized within the areas of tumor with CD31, a known blood vessel marker, while, again, no staining was seen in any normal brain tissue.

The same group then published in 2014 a PSMA immunohistochemistry study of 14 breast cancer patients with brain metastases [15]. They used a mouse 3E6 anti-PSMA antibody (Dako) for immunohistochemistry staining and quantified the extent of staining in tumor-associated vessels. The tumor vasculature of all brain metastases stained PSMA-positive, and the extent of expression was greater than 50%. They also scored the primary tumor for 10 of these patients, and in all cases, the primary tumor had greater than 50% expression. That same year, Nomura et al. published a PSMA tissue study of 23 gliomas and breast cancer brain metastases [16]. They used a mouse PSMA antibody mAb 3E6 (Dako) for immunohistochemistry and quantified the intensity of PSMA staining in tissue relative to a staining calibration curve normalized to mean image background intensity, using the automated algorithms in the Scanscope® CM-1 scanner and ImageScope® software. They found that GBM blood vessels stained heavily, grade II/III gliomas showed some (<2%) tumor tissue staining but no vessel staining, and grade I gliomas showed moderate vessel staining and some tumor staining. They additionally found that normal tissue blood vessels did not stain for PSMA but fewer than 5% of normal neurons did. The quantified relative intensity of staining in all gliomas was statistically significantly higher than in normal brain. They also reported corresponding PSMA and von Willebrand factor (VWF) staining on vascular endothelial cells. In brain metastases, they found variable staining within and between tumors, but on average the staining was significantly greater than normal brain.

**Figure 2:**
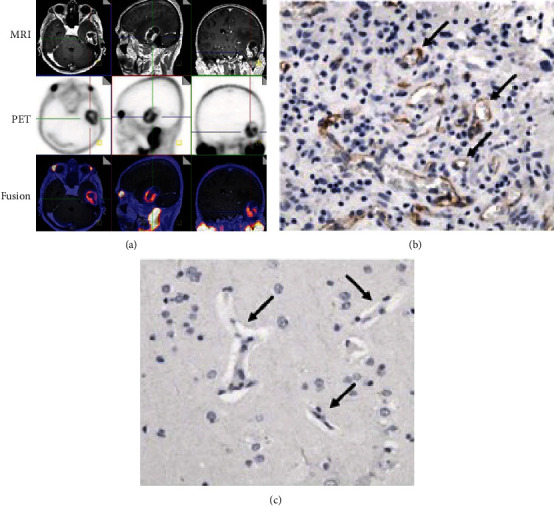
^68^Ga-PSMA PET and PSMA immunohistochemistry of representative GBM. (a) MRI, ^68^Ga-PSMA PET, and fused images. (b) PSMA expression in GBM vascular endothelium. (c) No PSMA expression in normal brain. Reproduced with permission from Schwenck et al. [17].

The above data were complemented by several subsequent case reports. Schwenck et al. reported increased PSMA expression in the vascular endothelium of a GBM patient, though not in the normal brain and vasculature (Figure 2) [17]. Subsequently, Unterrainer et al. reported the first PSMA study in a gliosarcoma patient, which demonstrated strong PSMA expression in the neovascular endothelial cells and not in the tumor tissue cells [18]. Salas Fragomeni et al. reported in one anaplastic astrocytoma and 2 GBMs that PSMA staining was localized to the vascular cells in GBM and tumor cells in anaplastic astrocytoma, and no staining was found in normal brain or vessels [19].

The two largest of these studies, by Matsuda et al. [20] and Saffar et al. [21] were both published in 2018 and demonstrated divergent results regarding the expression of PSMA in high-grade gliomas. Matsuda et al. studied tissue samples from 78 glioma, brain metastasis, CNS lymphoma, and radiation necrosis patients [20]. They used a rabbit monoclonal anti-PSMA antibody (EPR6253, Abcam) for immunohistochemistry, and they quantified the strength of tissue PSMA expression in vascular endothelial cells. They reported that PSMA was expressed in the vascular endothelial cells of almost all GBMs, most grade I and III gliomas, and all metastatic brain tumors. However, PSMA was expressed in few grade II gliomas and primary central nervous system lymphomas (PCNSL). They found no PSMA expression in radiation necrosis tissue. GBMs and brain metastases showed the highest expression levels, while few grade III gliomas and no grade II gliomas showed high expression. Saffar et al. reported on PSMA expression in 72 gliomas [21]. They used a monoclonal liquid Novocastra™ mouse monoclonal antibody (clone 1D6, Novocastra) for immunohistochemistry. They quantified the extent and intensity of PSMA staining in vascular endothelial cells. They found positive PSMA staining in a minority of gliomas, regardless of tumor grade. Among these, the GBMs and grade I gliomas had a lower extent of vascular staining, while the grade II and III gliomas had a higher extent of staining. The intensity of staining was weak for most of the GBMs and the grade III glioma, and it was moderate for the grade I and II gliomas.

The following year, Mahzouni et al. published a larger PSMA tissue study in GBMs (*n* = 60) [22]. They used an anti-PSMA mAB clone SP29 (Biogenex) for immunohistochemistry and quantified the extent and intensity of PSMA staining in vascular endothelial cells. They found PSMA staining in most GBMs, and among those, the extent of vascular staining was mostly 51-100% and the intensity of staining was mostly moderate or maximum.

Oliveira et al. then produced a preclinical model in which ^68^Ga-PSMA and ^18^F-DCFPyL binding were studied on ex vivo autoradiography in 38 rats, implanted with either F98, 9L, or U87 glioma cells [23]. They found that both PSMA-targeting tracers exhibited strong binding in the peritumoral area but moderate binding in the tumor core. In vivo animal PET imaging showed a higher tumor to background ratio (TBR) for ^18^F-DCFPyL (TBR 6.28-7.92) than ^68^Ga-PSMA (TBR 3.22-3.92). Tissue staining with three different anti-PSMA antibodies showed heterogeneous results, with one antibody negative for all three tumor cell lines (ab58779, Abcam), one positive in all three (NBP1-45057, Novus), and one split (NBP1-89822, Novus). PSMA-specific binding was confirmed by application of the PSMA-antagonist PMPA which effectively suppressed ^68^Ga-PSMA and ^18^F-DCFPyL binding. They found that activated microglia expression (CD11b) was low intratumorally and peritumorally but activated astrocyte expression (GFAP) was high peritumorally. They concluded that PSMA expression may truly be capturing astrocyte activation instead of tumorigenesis, which would limit its utility in differentiating tumor recurrence from radiation necrosis.

Recently, Liu et al. published a report from 30 glioma patients, in which they demonstrated PSMA expression on IHC staining in zero out of 14 grade II gliomas, two out of four grade III gliomas, and nine out of 12 GBMs [24]. Then, Holzgreve et al. demonstrated, in a series of 16 GBMs, using mouse mAb 3E6 (Agilent), that all 16 had PSMA uptake on IHC at initial diagnosis and 15/16 at recurrence [25]. The change in PSMA expression varied between these timepoints, whereas the vessel marker CD34 remained consistent. The level of vascular PSMA expression at recurrence was predictive of survival, as was an increase in PSMA expression over the course of the disease. Notably, PSMA expression was not associated with MGMT status or Ki-67 proliferation index.

In summary, tissue from multiple types of CNS tumors demonstrates PSMA expression. GBM and breast cancer brain metastases tissue showed robust PSMA staining, localized to the neovasculature, with staining present in a majority of cells with moderate to maximum intensity. PSMA staining metrics in GBM were also predictive of survival. Glioma tissue PSMA staining was highly variable across studies. No PSMA staining was seen in radiation necrosis human tissue; however, in glioma cell lines, there was evidence that PSMA expression may capture astrocyte activation instead of tumorigenesis.

### 3.2. PSMA-Targeting PET Tracer Properties

The properties of PSMA-targeting tracers differ on several key features, which results in different strategies for targeting PSMA [26]. For example, some tracers are small-molecule inhibitors while others are monoclonal antibodies [27]. The small-molecule tracers are smaller than antibody tracers and thus can travel faster throughout the vasculature and are excreted faster. Therefore, small-molecule tracers reach more tissue indiscriminately but allow for faster imaging protocols. Antibody tracers travel preferably through larger vessels, which are often seen more in tumor tissue than normal tissue. Therefore, antibody tracers allow for more specific imaging although the scanning protocol is longer. Both of these types of targeting compound are conjugated with a radionuclide to create a radiotracer targeting PSMA. There is variability between radionuclides, including their half-life and positron range. These tracer characteristics are described in detail below.

#### 3.2.1. Gallium-Tagged Tracers

Four formulations of ^68^gallium- (Ga-) tagged PSMA-targeting tracer have been developed, including ^68^Ga-PSMA-11 (HBED-CC), ^68^Ga-PSMA-617, ^68^Ga-PSMA-I&T,^28^ and ^68^Ga-THP-PSMA (Table 2) [28]. The ^68^Ga radionuclide is produced in a Ga generator, has a half-life of 1.1 hours, a positron range of 8.9 mm, and is taken up by the bladder wall and kidney [29, 30]. They do not show uptake in normal brain tissue. They may have a lower resolution than other isotopes, due to a longer photon range and the energy within the isotope.

^68^Ga-PSMA-11 (HBED-CC) benefits from a high affinity for PSMA-expressing tumors. It also shows rapid blood clearance and lower liver uptake, though it has uptake in salivary glands [29]. PSMA-617 can be labeled with ^177^Lu, ^255^Ac, or ^90^Y, for theranostic pairing, which gives it the potential to extend beyond the diagnostic sphere and into the treatment of PSMA-expressing lesions. ^68^Ga-PSMA-Imaging & Therapy (I&T) has high-affinity tumor uptake similar to HBED agents and also has theranostic pairing potential, as it can be labeled with ^177^Lu or ^255^Ac (Table 2). Among these tracers, only ^68^Ga-PSMA-11 (HBED-CC) and ^68^Ga-THP-PSMA have been studied in CNS tumors to date.

#### 3.2.2. Fluorine-Tagged Tracers

Three formulations of ^18^fluorine- (F-) tagged PSMA-targeting tracers have been developed, including ^18^F-DCFPyl [31], ^18^F-PSMA-1007 [31], and ^18^F-DCFBC [32]. These ^18^F radionuclides are produced in a cyclotron, have a half-life of 1.8 hours, a positron range of 0.6 mm, and are not taken up by normal brain tissue [30–32]. The route of excretion and critical uptake organs vary by tracer, and they include the liver, kidney, bladder wall, and gallbladder. These tracers all benefit from a better resolution than other isotopes, due to their short photon range. However, they all have significant salivary gland uptake.

^18^F-DCFPyL has a higher target-to-background ratio than ^68^Ga-PSMA-11 (HBED-CC) and thus may detect more lesions [29]. It has a much higher binding affinity for PSMA than its predecessor, ^18^F-DCFBC, which also suffered from high blood pool activity which limited detection of lymph nodes near blood vessels. ^18^F-PSMA-1007 is structurally similar to ^68^Ga-PSMA-617 but potentially has a better resolution [31]. When there are low PSA levels, subcentimeter bone lesions, lymph node involvement, or hepatic involvement, ^68^Ga-PSMA agents are superior to ^18^F-choline (Table 2). Among these, only ^18^F-DCFPyl and ^18^F-PSMA-1007 have been studied in CNS tumors to date.

#### 3.2.3. Copper-Tagged Tracers

One ^64^copper- (Cu-) tagged PSMA-targeting tracer was developed, and it was based on the structural base of ^68^Ga-PSMA-617 [33]. The ^64^Cu radionuclide is produced in a cyclotron, has a long half-life of 12.7 hours, a positron range of 0.6 mm, and accumulates in the liver, large intestine, and pancreas [30, 33]. It is not taken up by normal brain tissue. It benefits from theranostic pairing potential with ^67^Cu (Table 2). This radiotracer has not yet been studied in CNS tumors.

#### 3.2.4. Iodine-Tagged Tracers

There has been one ^124^iodine- (I-) tagged PSMA-targeting tracer developed, ^124^I-MIP-1095 [34]. The ^124^I radionuclide is produced in a cyclotron, has a long half-life of 100.8 hours, a positron range of 3.4 mm, and accumulates in the salivary glands, liver, and kidneys [30, 34]. It is not taken up by normal brain tissue. It benefits from theranostic pairing potential with ^131^I (Table 2). This radiotracer has not yet been studied in CNS tumors.

#### 3.2.5. Zirconium-Tagged Tracers

There has been one ^89^zirconium- (Zr-) tagged PSMA-targeting tracer developed, ^89^Zr-Df-IAB2M [35]. The ^89^Zr radionuclide is produced in a cyclotron, has a long half-life of 78.4 hours, a positron range of 1.2 mm, and accumulates in the liver and kidney [35, 36]. It is not taken up by normal brain tissue. This radiotracer has been studied in CNS tumors, but further clinical investigations are needed to understand its advantages and disadvantages relative to other tracers (Table 2).

#### 3.2.6. Technetium-Tagged Tracers

There have been five ^99m^technetium- (Tc-) tagged PSMA-targeting tracers developed, including Tc-PSMA [37], Tc-MIP-1404 [38], Tc-MIP-1405 [39], Tc-PSMA-I&S [40], and Tc-EDDA/HYNIC-iPSMA [41]. These radionuclides are produced by ^99m^Tc-generator and have a half-life of 6 hours [37–41]. The route of excretion and uptake by critical organs varies by tracer, but the kidneys are involved in all five, and none show uptake in normal brain tissue. All five Tc-tagged PSMA-targeting tracers have limited clinical application. As single-photon emission computerized tomography (SPECT) imaging tracers, they have a lower resolution than PET imaging tracers, although SPECT is more readily available in clinical settings than PET. Tc-PSMA-Imaging & Surgery (I&S) is being investigated for potential use in targeted surgery (Table 2). These radiotracers have not been studied in CNS tumors and are unlikely to be developed for this purpose given the superiority of PET over SPECT with regard to resolution.

### 3.3. Diagnostic Performance in Gliomas

#### 3.3.1. Gallium-PSMA in Gliomas

Recent years have seen an increase in PSMA-targeting PET imaging studies and case reports in gliomas, with a total of 20 studies with 122 patients, since 2015 (Table 3). Most of these studies have focused on imaging with the ^68^Ga-PSMA radiotracer, though a few have used alternative tracers such as ^18^F-DCFPyL, ^89^Zr-Df-IAB2M, and ^18^F-PSMA-1007. Following the promising results of early immunohistochemistry studies in glioma tissue, the first report of PSMA-targeting PET imaging of a glioma patient was published by Schwenck et al. [17]. They found that ^68^Ga-PSMA-11 (HBED-CC) uptake was markedly increased in the contrast-enhancing solid tumor region identified on MRI and that there was no uptake in the normal brain tissue (Figure 2). Then, Unterrainer et al. published the first case report of ^68^Ga-PSMA PET imaging in a gliosarcoma patient [18]. They found high ^68^Ga-PSMA-11 (HBED-CC) uptake, with a median maximal standardized uptake value (SUV_max_) of 3.43 and median maximal TBR (TBR_max_) of 48.93.

**Figure 3:**
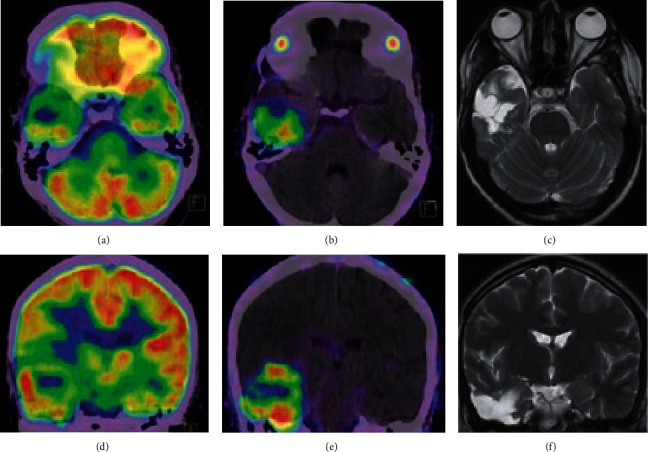
^18^F-FDG and ^68^Ga-PSMA PET imaging of representative GBM. (a) Axial ^18^F-FDG PET/CT. (b) Axial ^68^Ga-PSMA-11 PET/CT. (c) Axial T2-weighted MRI. (d) Coronal ^18^F-FDG PET/CT. (e) Coronal ^68^Ga-PSMA-11 PET/CT. (f) Coronal T2-weighted MRI. Reproduced with permission from Figure 1 in Sasikumar et al. [42].

The first case series of ^68^Ga-PSMA PET imaging in GBM was published by Sasikumar et al., which included five GBMs with suspected recurrence and one newly diagnosed GBM [42]. In four out of five GBMs with suspected recurrence, they found increased uptake of both ^68^Ga-PSMA-11 (HBED-CC) and ^18^F-FDG correlating to the lesion on MRI with histological confirmation. While the uptake of both tracers was spatially correlated, TBR was greater for ^68^Ga-PSMA-11 (HBED-CC) (12.9) than for ^18^F-FDG (0.96). The 5^th^ suspected recurrence, which did not demonstrate uptake with either tracer, did not have recurrence on histology. The newly diagnosed GBM showed intense tracer uptake on the periphery of the lesion, with a TBR of 22.3 for ^68^Ga-PSMA-11 (HBED-CC) and 1.11 for ^18^F-FDG. In this case series, they noted a better visualization of the lesion using ^68^Ga-PSMA-11 (HBED-CC) than ^18^F-FDG, which they attribute to its comparatively greater TBR. Sasikumar et al. later published a larger case series of 15 gliomas [43], which included the six GBMs from their 2017 report. In their 2018 report, they found that nine out of 10 suspected recurrences had positive findings on ^68^Ga-PSMA-11 (HBED-CC) PET scan, and the one without ^68^Ga-PSMA-11 (HBED-CC) uptake had no evidence of recurrence on further testing. The TBR values ranged from 4.07 to 29.4, compared to the patient with no disease whose TBR value was 1.15. They additionally found increased ^68^Ga-PSMA-11 (HBED-CC) uptake in two newly diagnosed GBMs and two postsurgical GBMs, but no uptake in a postsurgical grade III oligodendroglioma. Overall, in 13 ^68^Ga-PSMA-11 (HBED-CC) positive gliomas, the TBR was 34.78 in grade II, 11.9 and 27.0 in grade III, and 4.07-134.8 in grade IV. They concluded that the tracer uptake did not correlate to glioma grade (Figure 3).

A case report from Kunikowska et al. demonstrated high ^68^Ga-PSMA uptake in a GBM patient, with a SUV_max_ of 23.7 [44]. A report of ^68^Ga-PSMA PET imaging in an oligodendroglioma from Malik et al. [45] demonstrated increased ^68^Ga-PSMA uptake in the lesion and better lesion delineation with ^68^Ga-PSMA than with ^18^F-FDG. Verma et al. published a case series of 10 glioma patients, all of which demonstrated increased uptake on ^68^Ga-PSMA-11 (HBED-CC) PET imaging [46]. They found the SUV_max_ among GBM patients (16.93 ± 5.4) to be significantly higher than that of grade II gliomas (2.93 ± 0.3), as well as the TBR (13.95 versus 3.42), which runs contrary to the results of the Sasikumar et al. (2018) report.

Gupta et al. published a report of a recurrent GBM with ^68^Ga-PSMA-11 (HBED-CC) PET scan in the immediate postoperative period, which demonstrated residual ^68^Ga-PSMA-11 (HBED-CC) uptake in the margin of the postoperative cavity [47]. Gupta et al. (2020b) also published a report of increased ^68^Ga-PSMA-11 (HBED-CC) uptake on PET imaging of a posttreatment GBM with suspected recurrence, which was ultimately diagnosed as pseudoprogression based on subsequent imaging. The lesion SUV_max_ was 2.71, versus 0.52 in normal brain, and a TBR of 5.21. They warn that this false positive indicates that ^68^Ga-PSMA may not be fully able to differentiate recurrence from radiation necrosis. Moreau et al. echoed these concerns in their case report of a GBM patient with modest ^68^Ga-PSMA-11 (HBED-CC) uptake (SUV_max_ of 3.2), who was determined to have postradiation changes based on clinical imaging [48]. Without an established threshold for defining recurrence versus postradiation treatment effect, it may be difficult to interpret some of the ^68^Ga-PSMA PET images, especially given the wide range of values for SUV_max_ and TBR that have thus far been demonstrated in gliomas.

Pernthaler et al. report an oligodendroglioma patient with homogenously high ^68^Ga-PSMA-11 (HBED-CC) uptake and ^18^F-fluciclovine uptake, on PET imaging [49]. The SUV_max_ of ^68^Ga-PSMA-11 (HBED-CC) (9.7) was greater than that of ^18^F-fluciclovine (6.5). Pilati et al. reported another case of GBM with high ^68^Ga-PSMA-11 (HBED-CC) uptake on PET imaging [50]. Kumar et al. report a case of recurrent GBM with high ^68^Ga-PSMA-11 (HBED-CC) uptake on PET, which decreased as the tumor regressed posttherapy [51]. Zhang et al. (2020) report a glioma patient with heterogeneous ^68^Ga-PSMA uptake which was low in the core of the lesion and higher within foci along the edge of the lesion. On histopathology, it was found that the low uptake areas were grade II glioma tissue while the high uptake areas were grade III glioma tissue [52].

Kunikowska et al. published the largest series of GBMs imaged with ^68^Ga-PSMA-11 (HBED-CC) PET (*n* = 15) [53]. They found 100% spatial correlation of ^68^Ga-PSMA-11 (HBED-CC) uptake with MRI lesions, and 100% of the patients demonstrated increased ^68^Ga-PSMA-11 (HBED-CC) uptake, though the intensities and patterns differed. The median SUV_max_ was 6.5 (range 2.1-14.3), SUV_mean_ was 3.5 (range 1.3-6.1), and TBR was 96.7 (range 32.2-357.5). This TBR range is higher even than the range reported by Sasikumar et al. [43] which indicates a high degree of differentiation between tumor and normal brain. These values are also higher than the reported TBRs of amino acid PET tracers like ^18^F-FET and ^11^C-MET PET [54], which demonstrates that ^68^Ga-PSMA better differentiates lesions from the background. Kunikowska et al. also investigated the tumor-to-liver (TLR) ratio because a TLR of 1.5 or higher is needed to be eligible for current PSMA-based radionuclide targeted therapy. They found the median TLR was 0.8 (range 0.6-1.8), and only 2/15 (13%) patients had a TLR of 1.5 or higher.

The largest glioma case series imaged with ^68^Ga-PSMA PET (*n* = 35) was published by Akgun et al., in which they found tumor grade to be moderately correlated with SUV_max_ (*r* = 0.53), SUV_mean_ (*r* = 0.55), and SUV_peak_ (*r* = 0.50) [55]. Grade II and III glioma SUV values were each significantly different from grade IV gliomas, though grade II and III were not significantly different from each other. To differentiate grade II/III from grade IV, the SUV_max_ cutoff was 2.3 (sensitivity 80%, specificity 81.8%, PPV 81.5%, NPV 80.3%). To differentiate low-grade glioma (LGG; grade II) from high-grade glioma (HGG; grades III/IV), the SUV_max_ cutoff was 1.15 (sensitivity 85.7%, specificity 85.7%, PPV 85.7%, NPV 85.7%), which compared favorably to MRI (sensitivity 71.4%, specificity 85.4%) in diagnosing HGG. ^68^Ga-PSMA PET was significantly more sensitive (*p* < 0.05) than MRI, but not more specific. They also found that Ki-67, an immunohistochemical proliferation index, was moderately correlated with SUV_max_ (*r* = 0.51), SUV_mean_ (*r* = 0.48), and SUV_peak_ (*r* = 0.44). Mitosis was highly correlated with SUV_max_ (*r* = 0.64) and moderately correlated with SUV_mean_ (*r* = 0.58) and SUV_peak_ (*r* = 0.56). Endothelial proliferation was moderately correlated with SUV_mean_ (*r* = 0.40). Necrosis was moderately correlated with SUV_max_ (*r* = 0.48), SUV_mean_ (*r* = 0.56), and SUV_peak_ (*r* = 0.49). ATRX mutation status was not significantly correlated to any SUV value.

In a study of 30 pathology-confirmed glioma patients, Liu et al. found that SUV_max_ and SUV_mean_ were higher for ^68^Ga-PSMA PET (AUC 0.96 and 0.94) than for FDG PET (AUC 0.79, 0.74) [24]. ^68^Ga-PSMA PET was more effective than FDG PET for distinguishing HGG from LGG. ^68^Ga-PSMA SUV_max_ for LGG was 0.74 and for HGG was 5.8, while FDG SUV_max_ was 9.2 for LGG and 11.8 for HGG. With a cutoff of 2.21, ^68^Ga-PSMA SUV_max_ achieved sensitivity 0.81, specificity 1.00, and *p* < 0.001, while with a cutoff of 10.41, FDG SUV_max_ achieved sensitivity 0.69, specificity 0.86, and *p* = 0.0083.

#### 3.3.2. ^18^F-DCFPyL, ^89^Zr-Df-IAB2M, and ^18^F-PSMA-1007 in Gliomas

Although most PSMA-targeting PET imaging studies in glioma have been done with the ^68^Ga-PSMA tracer, there have been a few studies with alternate tracers. Salas Fragomeni et al. evaluated ^18^F-DCFPyL PET in 3 HGGs, which all showed increased ^18^F-DCFPyL uptake [19]. The SUV_max_ ranged from 5.8 to 13.5 in tumor lesions, and no uptake was seen in normal brain tissue. Matsuda et al. used ^89^Zr-Df-IAB2M PET to image 2 HGGs [20]. One HGG showed high uptake in the areas of robust contrast enhancement and low uptake in the areas of lesser enhancement. The other HGG showed high but heterogeneous uptake within the lesion, but with a different distribution pattern than ^11^C-MET uptake. Marafi et al. evaluated 1 recurrent GBM patient with ^18^F-PSMA-1007 PET [56]. This patient showed increased uptake of both ^18^F-PSMA-1007 and ^18^F-FDG in the area of the lesion on MRI, though the differential uptake of ^18^F-PSMA-1007 in tumor versus normal brain was better than ^18^F-FDG, so the lesion was more clearly delineated with ^18^F-PSMA-1007.

#### 3.3.3. PSMA in Gliomas Summary

In summary, ^68^Ga-tagged PSMA-targeting PET showed robust uptake in gliomas and GBMs, with no uptake in normal brain regions. Across glioma grades, these tracers showed greater uptake than ^18^F-FDG. There is conflicting data about whether these uptake values are correlated with tumor grade, but SUV_max_ cutoff of 2.3 has been proposed for differentiating grade II/III and IV gliomas and SUV_max_ 1.15 or 2.21 for LGG and HGG. Radiation necrosis also demonstrated increased uptake, which calls into question whether this imaging modality can be used to differentiate progression from pseudoprogression. Little research has been done on ^18^F- and ^89^Zr-tagged PSMA-targeting tracers.

### 3.4. Diagnostic Performance in Nonglioma CNS Tumors

#### 3.4.1. Brain Metastases from Prostate Cancer

Metastases to the brain from prostate cancer are rare, with few reported cases in the literature. There are 10 published case reports (*n* = 18 patients) of ^68^Ga-PSMA PET imaging of such metastases (Table 4). In nearly all cases, these were detected incidentally on ^68^Ga-PSMA screening for metastases.

In some reports, the diagnosis of prostate cancer metastasis was made using MRI. The first was published in 2015 by Chakraborty et al., in which they reported a prostatic adenocarcinoma patient without neurologic symptoms, who demonstrated avid ^68^Ga-PSMA-11 (HBED-CC) uptake in three separate MRI-diagnosed brain metastases, in the cerebellar hemisphere and bifrontal lobes [57]. Dureja et al. reported a prostate adenocarcinoma patient without neurologic symptoms who was found on ^68^Ga-PSMA-11 (HBED-CC) PET imaging to have at least four MRI-diagnosed brain metastases in the frontal and frontoparietal lobes [58]. The SUV_max_ of the largest lesion was 4.0. Langsteger et al. reported the only MRI-diagnosed spinal cord metastasis from prostate cancer detected with ^68^Ga-PSMA PET imaging [59]. The patient had no neurologic symptoms but was found to have ^68^Ga-PSMA uptake in the spinal cord at the T4-T10 levels.

In other reports, the diagnosis was confirmed by histopathology. Chan et al. reported a patient with prostate cancer who presented with mild ataxia and nausea and upon imaging with ^68^Ga-PSMA-11 (HBED-CC) was found to have a single metastasis in the cerebellum [60]. The ^68^Ga-PSMA-11 (HBED-CC) uptake in the lesion was SUV_max_ 5.9, and diagnosis was confirmed by histopathology consistent with high-grade metastatic prostate adenocarcinoma with positive staining for PSMA. Ross et al. reported an asymptomatic temporal lobe metastasis which was not detected on conventional imaging, but was found on ^68^Ga-THP-PSMA PET imaging and was confirmed with histopathology to be prostate adenocarcinoma [61]. Yin et al. (2019) reported a patient with prostate adenocarcinoma without neurologic symptoms who was found incidentally to have temporal and occipital metastatic lesions on ^68^Ga-PSMA PET [62]. The lesions demonstrated ^68^Ga-PSMA avidity with SUV_max_ ranging 3-11, and not all of the metastases were detected on MRI. Histopathology confirmed the diagnosis and demonstrated positive staining for PSA. Of note, this is reported to be the longest surviving prostate cancer patient with multiple brain metastases at three years, which the authors attribute to the early detection with ^68^Ga-PSMA PET.

In two reports, the application of PSMA therapeutics reduced both the size of the metastases and the ^68^Ga-PSMA uptake on PET imaging. Wei et al. reported two prostate cancer patients with known brain metastases who underwent ^68^Ga-PSMA-11 (HBED-CC) PET imaging for restaging and demonstrated distinct ^68^Ga-PSMA-11 (HBED-CC) uptake in the cerebral lesions [63]. The ^68^Ga-PSMA-11 (HBED-CC) uptake in these two patients decreased as the size of the lesions regressed, following combined ^177^Lu-PSMA-617 with radiotherapy. Sathekge et al. reported one castration-resistant prostate cancer patient with several cerebral metastases that showed avid ^68^Ga-PSMA uptake on PET imaging [64]. Following treatment with ^225^Ac-PSMA-617, the size and ^68^Ga-PSMA uptake of the cerebral metastases decreased, along with the PSA levels. On the other hand, Parihar et al. reported a case of new brain metastases with ^68^Ga-PSMA uptake, after treatment of prostate cancer with ^177^Lu-PSMA and ^225^Ac-PSMA [65]. This patient was treated with docetaxel, cabazitaxel, enzalutamide, and two cycles of ^177^Lu-PSMA therapy, and then four cycles of ^225^Ac-PSMA therapy. After two cycles of ^225^Ac-PSMA therapy, ^68^Ga-PSMA PET/CT imaging did not demonstrate any brain lesion, and then after four total cycles of ^225^Ac-PSMA, imaging revealed new brain lesions in the setting of generalized disease progression.

The first extended case series of PSMA-targeting PET in prostate cancer brain metastasis (n =8), by McBean et al., revealed considerable variability across patients [66]. Only three out of eight patients presented with neurological symptoms, and the ^68^Ga-PSMA avidity ranged from not avid to intense, while the SUV_max_ ranged from 2 to 21. Of note, four out of eight patients had previously undergone ^177^Lu-PSMA therapy for their primary prostate cancer, but this did not correlate with any trends in their imaging findings.

#### 3.4.2. Brain Metastases from Nonprostate Cancer

**Figure 4:**
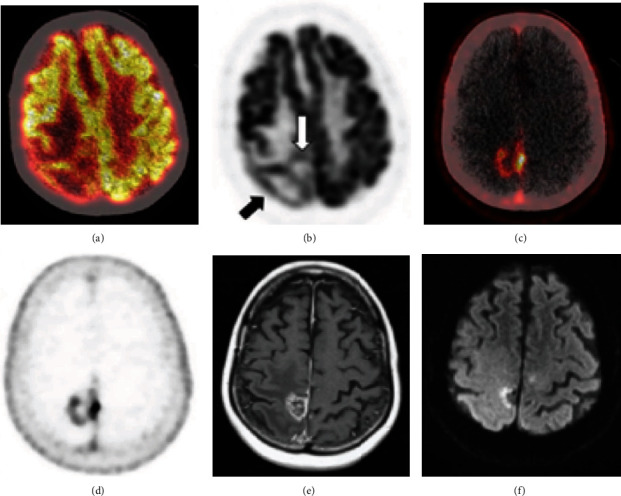
^18^F-FDG and ^18^F-PSMA PET MRI imaging of representative breast cancer brain metastasis. (a) ^18^F-FDG PET/CT. (b) ^18^F-FDG PET. (c) ^18^F-PSMA-1007 PET/CT. (d) ^18^F-PSMA-1007 PET. (e) T1-weighted MRI. (f) DWI MRI. Reproduced with permission from Figure 1 in Marafi et al. [68].

There has also been a selection of case reports of brain metastases that arise from nonprostate cancers that nevertheless demonstrate significant PSMA-targeted tracer uptake on PET (Table 5). Four such reports of PSMA-ligand uptake in brain metastases from breast cancer patients have been published. Medina-Ornelas et al. reported a patient with HER-2-positive breast carcinoma, who presented with progressive headache and dizziness, and was subsequently found to have a supratentorial brain metastasis on MRI and ^68^Ga-PSMA PET, confirmed by histopathology [67]. Malik et al. reported a breast cancer patient with a cerebellar brain metastasis that avidly accumulated ^68^Ga-PSMA, but not ^18^F-FDG [45]. Marafi et al. also reported a breast cancer patient with intense ^18^F-PSMA-1007 uptake in several MRI-diagnosed metastases in the parietal lobe and cerebellum, without uptake in normal tissue (Figure 4) [68]. Arslan et al. reported a triple-negative breast cancer patient with a recurrent brain metastasis that demonstrated avid ^68^Ga-PSMA-11 (HBED-CC) uptake, but low FDG [69]. Diagnosis of this frontoparietal lesion was confirmed by histopathology.

Two reports have been published of ^18^F-DCFPyL uptake on PET in metastatic renal cell carcinomas (RCC), one clear cell and one nonclear cell. Rowe et al. reported a clear cell RCC patient who demonstrated intense ^18^F-DCFPyL uptake in an MRI-diagnosed frontal lobe brain metastasis (SUV_max_ = 3.9) [70]. Yin et al. reported a nonclear cell RCC patient with three CT-diagnosed brain metastases, in the parietal and temporal lobes, with ^18^F-DCFPyL SUV_max_ ranging from 0.5 to 3.4 [71].

There is only one report of ^68^Ga-PSMA uptake in a brain metastasis from melanoma. Hod et al. reported a patient who demonstrated a melanoma metastasis in the occipital lobe on CT and MRI and unexpectedly showed avid ^68^Ga-PSMA uptake in the lesion [72]. Diagnosis was confirmed by histopathology.

Similarly, there has been only one report of ^89^Zr-Df-IAB2M uptake in brain metastases from lung cancer. Matsuda et al. report a lung cancer patient with MRI-detected occipital brain metastasis, which demonstrated avid ^89^Zr-Df-IAB2M uptake [20]. There was no uptake in normal tissue. Diagnosis was confirmed by histopathology, and immunohistochemical analysis demonstrated a trend toward a positive correlation between ^89^Zr-Df-IAB2M uptake and PSMA expression.

#### 3.4.3. Meningiomas

**Figure 5:**
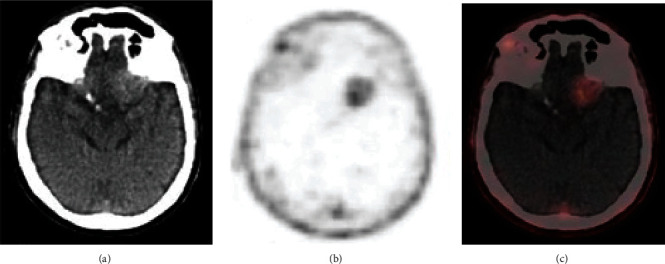
^68^Ga-PSMA PET/CT in a representative meningioma. (a) T1-weighted MRI. (b) Head CT. (c) ^68^Ga-PSMA PET/CT. Reproduced with permission from Figure 2 of Bilgin et al. [73].

PSMA targeting is potentially well suited to meningiomas, given that these lesions are generally highly vascularized. There have been eight single case reports published of PSMA-targeted PET imaging in meningioma patients, all of which were incidental findings in patients with prostate cancer (Table 6). Often the lesions were suspected brain metastases that were ultimately diagnosed as meningiomas. In six of these reports, the PSMA-binding tracer utilized was ^68^Ga-PSMA. Bilgin et al. reported a patient with high ^68^Ga-PSMA uptake in an orbitofrontal lesion (SUV_max_ = 3.1) which was subsequently diagnosed as meningioma on MRI [73] (Figure 5). Another report, by Jain et al., found that a frontal convexity meningioma demonstrated avid ^68^Ga-PSMA uptake (SUV_max_ = 1.9), but no FDG avidity [74]. Sasikumar et al. reported a patient with an ambiguous lesion on MRI, which demonstrated stronger ^68^Ga-PSMA-11 (HBED-CC) uptake (SUV_max_ lesion 14.6, SUV_max_ background 0.5, TBR 29.2) than ^18^F-FDG uptake (SUV_max_ lesion 6.44, SUV_max_ background 8.62, TBR 0.74) [42]. Histopathology confirmed a diagnosis of atypical meningioma. Gupta et al. also reported a parietal lobe meningioma that demonstrated avid ^68^Ga-PSMA uptake, and diagnosis was supported by MRI [75]. Courtney et al. reported a patient with a history of prostate cancer who demonstrated ^68^Ga-PSMA uptake in the left frontal lobe, confirmed as a preexisting meningioma [76]. Finally, Junqueira et al. reported avid ^68^Ga-PSMA uptake (SUV_max_ 12.1) in an MRI-detected lesion, suggestive of an intraventricular meningioma of a patient with history of prostate cancer [77].

One report utilized the ^64^Cu-PSMA tracer. Calabria et al. reported an MRI-detected foramen magnum meningioma with SUV_max_ of 3.8 at 1 hour after tracer administration and 3.9 at four hours postadministration [78].

Similarly, one report utilized the ^18^F-PSMA-1007 tracer. Haemels et al. reported a patient with moderately avid ^18^F-PSMA-1007 uptake in the occipital lobe [79]. This was diagnosed by histopathology as a transitional-type meningioma. Tissue autoradiography studies revealed specific ^18^F-PSMA-1007 binding, which could be inhibited by 2-PMPA.

#### 3.4.4. Other CNS Tumors

There are an additional three case reports (with *n* = 4 patients) of ^68^Ga-PSMA PET imaging in other CNS tumors, including CNS lymphoma, intracranial hemangiopericytoma, and hemangioblastoma. Sasikumar et al. reported two patients with histopathology-confirmed CNS lymphoma [42]. They both demonstrated higher ^68^Ga-PSMA-11 (HBED-CC) uptake than ^18^F-FDG uptake: in the first patient, ^68^Ga-PSMA-11 (HBED-CC) uptake showed TBR of 17.0 (SUV_max_ lesion 3.07, SUV_max_ background 0.18), and ^18^F-FDG uptake showed TBR 4.85 (SUV_max_ lesion 28.7, SUV_max_ background 5.91, TBR); in the second, ^68^Ga-PSMA-11 (HBED-CC) uptake showed TBR of 37.2, versus 3.6 for ^18^F-FDG. Patro et al. reported a patient with an intracranial hemangiopericytoma in the posterior fossa, which demonstrated intense ^68^Ga-PSMA-11 (HBED-CC) uptake and mild ^18^F-FDG enhancement [80]. Gohimont et al. reported a patient with a history of prostate cancer, who demonstrated ^68^Ga-PSMA-HBED-CC uptake in the cerebellum on PET/CT, which was confirmed histopathologically as a hemangioblastoma [81].

#### 3.4.5. PSMA in Other CNS Tumors Summary

In summary, PSMA-targeting PET showed uptake in prostate cancer CNS metastases with variable avidity. Only case reports and small case series have been published though, and there is a need for systematic study of this imaging modality in this patient population. Anecdotal PSMA-targeted PET imaging has also shown uptake in brain metastases from breast cancer, RCC brain, melanoma, and lung cancer. There are also a few reports of ^68^Ga-tagged PSMA-targeted tracer uptake in meningiomas, though more research is needed to understand the potential use of this imaging modality in meningiomas.

### 3.5. Potential Therapeutic and Theragnostic Applications of PSMA

#### 3.5.1. Labeled Alpha-Ray Emitting Radioisotopes

**Figure 6:**
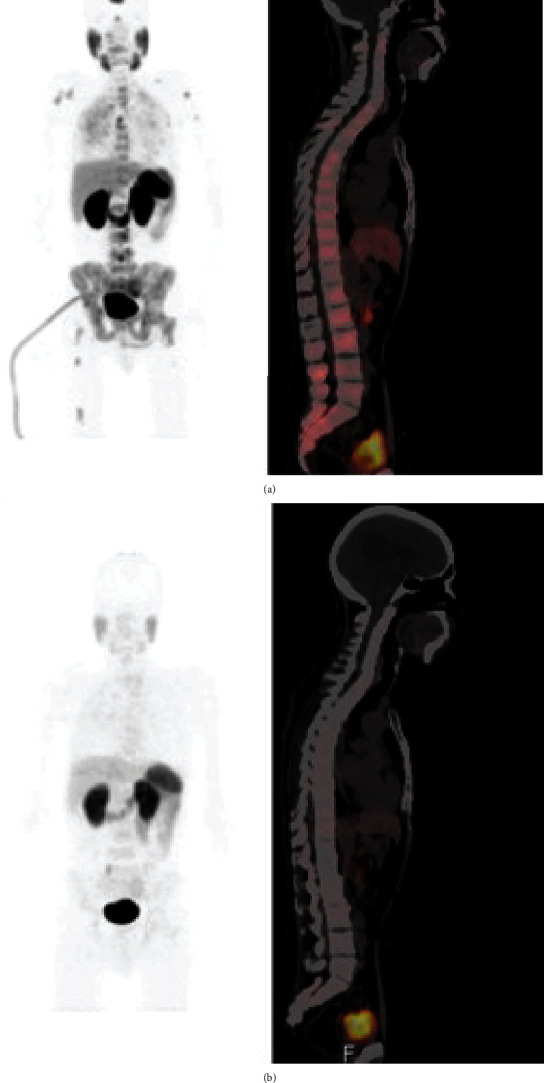
Response to ^225^Ac-PSMA-617 therapy in a representative prostate cancer brain metastasis. (a) Pretreatment ^68^Ga-PSMA PET/CT. (b) Restaging PSMA PET/CT scan after one cycle of ^225^Ac-PSMA. Reproduced with permission from Sathekge et al. [64].

^225^Ac-PSMA-617 is an alpha-emitting radioisotope-labeled derivative of PSMA-617, which targets the overexpressed PSMA seen in prostate cancer, and this therapy has demonstrated excellent therapeutic response in prostate cancer [82, 83]. It was also recently noted to be effective in the treatment of prostate cancer brain metastases [64]. Initial assessment with ^68^Ga-PSMA PET revealed cerebral metastases, and then, following one and two cycles of 8 MBq of ^225^Ac-PSMA-617 treatment, there was a functional response on ^68^Ga-PSMA PET. The serum PSA level dropped from an initial 788.63 *μ*g/L to 6.52 *μ*g/L, to 0.32 *μ*g/L. This incidental finding demonstrates the potential for this prostate cancer therapy to be applied in brain metastases and perhaps other PSMA-expressing CNS tumors as well (Figure 6).

#### 3.5.2. Labeled Beta-Ray Emitting Radioisotopes

^177^Lu is a beta-emitting radioisotope, and ^177^Lu-PSMA-617 targets overexpressed PSMA and has demonstrated excellent therapeutic response in metastatic prostate cancer [84]. The first report of ^177^Lu-PSMA-617 therapy use in prostate cancer brain metastases came from Wei et al., in which they report significant regression of cerebral lesions following ^177^Lu-PSMA-617 treatment plus local radiotherapy in two patients [63]. The first had several radiation-refractory cerebral and cerebellar metastases, which showed ^68^Ga-PSMA-11 (HBED-CC) uptake on initial PET imaging. After four cycles of ^177^Lu-PSMA-617 therapy totaling 25.5 GBq, the lesions demonstrated reduced size and PSMA expression. This correlated to a decrease in serum PSA from 195 ng/mL to 2.4 ng/mL. The second patient had one brain metastasis that was diagnosed after several hormonal and chemotherapy systemic treatments. This lesion showed ^68^Ga-PSMA-11 (HBED-CC) uptake on initial PET, and after three cycles of ^177^Lu-PSMA-617 therapy, the first of which was combined with radiotherapy; it had nearly completely regressed. This was accompanied by a decrease in serum PSA from 112 ng/mL to 3.9 ng/mL. Recently, Kumar et al. reported on the successful application of ^177^Lu-PSMA-617 therapy in a glioblastoma patient [51]. The patient presented with recurrent disease, which demonstrated intense ^68^Ga-PSMA-11 (HBED-CC) uptake in the MRI-enhancing areas. The patient underwent three cycles of ^177^Lu-PSMA-617 for a total dose of 3700 MBq, after which MRI and ^68^Ga-PSMA-11 (HBED-CC) PET demonstrated a reduction in lesion size (from 18 mL to 5.4 mL), which was accompanied by an improvement in ECOG score from 4 to 3. These positive results are tempered by a recent report from Parihar et al., which showed the emergence of new brain metastasis with ^68^Ga-PSMA uptake, after ^177^Lu-PSMA and ^225^Ac-PSMA treatment of prostate cancer [65].

#### 3.5.3. MRI-Guided Focused Ultrasound

MRI-guided focused ultrasound (MRgFUS) has been leveraged as a tool for disrupting the blood-brain barrier (BBB) in order to permit the passage of drugs, for a number of CNS indications [85]. In a preclinical study of nine rats with nondiseased brains, Airan et al. demonstrated that ^18^F-DCFPyL could be administered and targeted to a specific brain focus using MRgFUS for BBB disruption [86]. Following MRgFUS, ^18^F-DCFPyL uptake localized to the foci of BBB disruption and extended minimally beyond the contrast-enhancing MRI lesion. Target uptake plateaued at 80 minutes postadministration, with a maximal TBR of 7. PSMA-binding specificity was confirmed by administering the anti-PSMA ZJ-43 which lowered TBR fourfold. Although this was performed on healthy brains, it demonstrated the possibility of leveraging MRgFUS for opening the BBB to allow hydrophilic ^18^F-DCFPyL PSMA to enter and reach target tissue within the brain.

## 4. Future Objectives

The PSMA-binding tracer mechanism of expression is thought to be specific to tumor vasculature, not to invading or proliferating cells. While this may limit its utility as an isolated imaging modality, it may prove to be a useful adjunct to other neuroimaging modalities for CNS tumors. On the other hand, the significant potential advantage of PSMA-targeted PET tracers is in CNS tumor theranostics, which the current literature only begins to describe. There may be the potential for a wide range of future applications, such as small molecule selective PSMA inhibitors and radiosensitizers. Pairing PSMA-targeted diagnostic imaging with PSMA-targeted therapeutics may yield a robust theragnostic option for CNS tumor patients.

## 5. Conclusions

In summary, this systematic review of the literature on PSMA-targeted tracer use in CNS tumors demonstrates promising results about the potential for this PET tracer for neuro-oncologic imaging. Future efforts should continue to explore the potential for PSMA in neuro-oncologic imaging, specifically focusing on proposed mechanism(s) of PSMA expression in CNS tumors, differential imaging performance by CNS tumor type, direct comparison of PSMA-targeted PET to other neuroimaging modalities for diagnosing and monitoring CNS tumors, and direct comparison of competing PSMA-binding radiotracers. The surreptitious discovery of the utility of PSMA-targeted PET imaging in CNS tumors has invigorated much excitement in the field about its potential clinical application, but more research is needed to better understand this PET tracer before it can be implemented clinically.

## Figures and Tables

**Table 1:** Summary of tissue expression of PSMA.

Authors	Year	*n*	Tumor type	Tissue stain	Main results
Chang et al.	1999	1	GBM	7E11 (Cytogen) J591 (homemade) J415 (homemade) PEQ226.5 (Hybritech) PM2J004.5 (Hybritech)	(i) All 5 anti-PSMA monoclonal antibodies react strongly with the neovasculature of GBM (ii) No expression in tumor cells or normal brain tissue
Wernicke et al.	2011	32	GBM	Mouse Ab 3E6 (Dako)	(i) 32/32 specimens stained for PSMA (ii) 7/32 had 75-100% vascular staining, 15/32 had 51-75% staining, 2/32 had 26-50% staining, and 8/32 had 1-25% staining (iii) 14/32 had 3+ staining intensity, 15/32 had 2+ intensity, and 3/32 had 1+ intensity (iv) No expression in the vessels of normal brain
Wernicke et al.	2014	14	Breast cancer brain metastases	Mouse Ab 3E6 (Dako)	(i) 14/14 had PSMA-positive expression in metastasis vasculature (ii) 14/14 had staining intensity of 2 (PSMA-positive in 50%+ tumor vessels)
Nomura et al.	2014	23	5 grade I gliomas 4 grade II gliomas 5 grade III gliomas 5 GBMs 4 breast cancer brain metastases	Mouse Ab 3E6 (Dako)	(i) Grade I gliomas: moderate tumor vascular staining, some tumor cell staining (ii) Grade II/III gliomas: light staining of some tumor cells (<2%), little/no vascular staining (iii) GBMs: heavy tumor vascular staining (iv) Breast cancer brain metastases: vascular staining, less than primary lesion, variable intensity within tumor (v) No expression in normal brain vasculature (vi) <5% expression in normal brain tissue
Schwenck et al.	2015	1	GBM	Unknown	(i) PSMA expression predominantly in tumor vascular endothelial cells (ii) No PSMA expression in normal brain tissue or vasculature
Unterrainer et al.	2017	1	Gliosarcoma	Unknown	(i) Strong PSMA expression in tumor vascular endothelial cells (ii) No expression in tumor tissue
Salas Fragomeni et al.	2017	3	1 anaplastic astrocytoma 2 GBMs	Unknown	(i) Anaplastic astrocytoma: PSMA expression localized to tumor cells (ii) GBMs: PSMA expression localized to tumor vasculature (iii) No expression in normal brain tissue or vasculature
Saffar et al.	2018	72	10 grade I gliomas 26 grade II gliomas 9 grade III gliomas 27 GBMs	Mouse Ab 1D6 (Novocastra)	(i) Grade I gliomas: 2/10 had PSMA expression, with staining intensity of 2; 8/10 had 0% extent of staining, 2 had 10-39% extent (ii) Grade II gliomas: 1/26 had PSMA expression, with staining intensity of 25/26 had 0% extent of staining, 1 had >70% extent (iii) Grade III gliomas: 1/9 had PSMA expression, with staining intensity of 1; 8/9 had 0% extent of staining, 1/9 had >70% extent (iv) GBMs: 11/27 had PSMA expression; 9/27 had staining intensity of 1, 1/27 had intensity of 2, 1/27 had intensity of 3; 16/27 had 0% extent of staining, 4/27 had 1-9% extent, 5/27 had 10-39% extent, 1/27 had 40-69% extent, 1/27 had >70% extent
Matsuda et al.	2018	78	4 grade I gliomas 7 grade II gliomas 15 grade III gliomas 41 GBMs 4 brain metastases 7 PCNSLs	Rabbit mAb EPR6253 (Abcam)	(i) Grade I gliomas: 3/4 had PSMA expression (ii) Grade II gliomas: 1/7 had PSMA expression; 0/7 with high expression (iii) Grade III gliomas: 10/15 had PSMA expression; 5/15 with high expression (iv) GBMs: 40/41 had PSMA expression; 32/41 with high expression (v) Brain metastases: 4/4 had PSMA expression; 3/4 with high expression (vi) PCNSLs: 2/7 had PSMA expression (vii) Radiation necrosis: 0/5 had PSMA expression
Mahzouni et al.	2019	60	GBM	Rabbit mAb SP29 (Biogenex)	(i) 40/60 with PSMA expression in tumor vasculature (ii) 3/60 with 76-100% extent of staining, 16/60 with 51-76% extent, 9/60 with 26-50% extent, 12/60 with 6-25% extent, 20/60 with <5% extent (iii) 15/60 with severe intensity of staining, 23/60 with moderate intensity, 7/60 with mild intensity, 15 with zero intensity (iv) No staining of normal brain vasculature
Oliveira et al.	2020	38^∗^	Rat glioma (with F98, 9L, or U87 cells)	ab58779 (Abcam) NBP1-89822 (Novus) NBP1-45057 (Novus)	(i) Both 68Ga-PSMA and 18F-DCFPyL were expressed more in the peritumoral area than the tumor core, on ex vivo autoradiography (ii) Higher TBR for ^18^F-DCFPyL (TBR 6.28-7.92) than ^68^Ga-PSMA (TBR 3.22-3.92) on PET (iii) Heterogeneous tissue staining with 3 PSMA antibodies: ab58779 (Abcam) negative ×3, NBP1-45057 (Novus) positive ×3, and NBP1-89822 (Novus) split (iv) Activated astrocyte expression (GFAP) was high peritumorally
Liu et al.	2021	30	14 grade II gliomas 4 grade III gliomas 12 GBMs	Unknown	(i) PSMA-positive IHC staining in 0/14 grade II, 2/4 grade III, and 9/12 grade IV
Holzgreve et al.	2021	16	GBM	Mouse mAb 3E6 (Agilent)	(i) PSMA expression in all GBMs at initial diagnosis and 15/16 at recurrence (ii) Variable temporal evolution of PSMA expression from diagnosis to recurrence (iii) High vascular PSMA expression at recurrence inversely associated with survival (iv) Increasing PSMA expression over disease course inversely associated with survival (v) No correlation between PSMA expression and MGMT or Ki-67

**Table 2:** Summary of PSMA radiotracer properties.

PSMA radiotracer	Compound structure	Radionuclide synthesis	Radionuclide half-life (hours)	Positron range (mm)	Tracer excretion	Critical organ(s)	Brain uptake	Advantages	Disadvantages
^68^Ga-PSMA-11 (HBED-CC) [27, 29]	Small molecule	Ga generator	1.1	8.9	Renal	Bladder wall Kidney	No	(i) High affinity for PSMA-expressing tumors (ii) Rapid blood clearance, low liver uptake	(i) Decreased resolution because of longer photon range (ii) Uptake in salivary glands
^68^Ga-PSMA-617 ^28, 30^	Small molecule	Ga generator	1.1	8.9	Renal	Bladder wall Kidney	No	(i) Potential theranostic pairing with ^177^Lu, ^255^Ac, or ^99^Y	(i) Decreased resolution because of longer photon range (ii) Uptake in salivary glands
^68^Ga-PSMA-I&T ^27, 28, 30^	Small molecule	Ga generator	1.1	8.9	Renal	Bladder wall Kidney	No	(i) High affinity for PSMA-expressing tumors (ii) Potential theranostic pairing with ^177^Lu or ^255^Ac	(i) Decreased resolution because of longer photon range (ii) Uptake in salivary glands
^68^Ga-THP-PSMA [27, 28, 30]	Small molecule	Ga generator	1.1	8.9	Renal	Bladder wall Kidney	No	(i) Production can be done as a one-step process using a kit	(i) Decreased resolution because of longer photon range
^18^F-DCFPyL [27, 30, 31]	Small molecule	Cyclotron	1.8	0.6	Renal	Bladder wall Kidney	No	(i) Better resolution because of shorter photon range (ii) Higher binding affinity for PSMA	(i) Significant salivary gland accumulation
^18^F-PSMA-1007 ^27, 30, 31^	Small molecule	Cyclotron	1.8	0.6	Hepatobiliary	Gallbladder Liver	No	(i) Better resolution because of shorter photon range	(i) Significant salivary gland accumulation
^18^F-DCFBC [27, 30, 32]	Small molecule	Cyclotron	1.8	0.6	Renal	Bladder wall Kidney	No	(i) Better resolution because of shorter photon range	(i) Significant salivary gland accumulation (ii) High blood pool activity
^64^Cu-PSMA [27, 33, 36]	Antibody	Cyclotron	12.7	0.6	Biliary	Large intestine Liver Pancreas	No	(i) Potential theranostic pairing with ^67^Cu	(i) Low percentage of positron emission
^124^I-MIP-1095 ^30, 34^	Small molecule	Cyclotron	100.8	3.4	Hepatobiliary	Kidney Liver Salivary gland	No	(i) Potential theranostic pairing with ^131^I	(i) Access to I^124^ is challenging
^89^Zr-Df-IAB2M [35, 36]	Antibody	Cyclotron	78.4	1.2	Hepatobiliary	Kidney Liver	No	(i) Unknown	(i) Little clinical experience
^99m^Tc-PSMA [37, 87]	Small molecule	Tc generator	6	n/a	Renal	Kidney Liver	No	(i) SPECT more readily available than PET	(i) Little clinical experience (ii) SPECT lesser resolution and less sensitive than PET
^99m^ Tc-MIP-1404 ^27, 38^	Small molecule	Tc generator	6	n/a	Renal	Kidney Liver Salivary gland	No	(i) SPECT more readily available than PET	(i) Little clinical experience (ii) SPECT lesser resolution and less sensitive than PET
^99m^ Tc-MIP-1405 ^27, 39^	Small molecule	Tc generator	6	n/a	Renal	Kidney Liver Salivary gland	No	(i) SPECT more readily available than PET	(i) Little clinical experience (ii) SPECT lesser resolution and less sensitive than PET
^99m^ Tc-MIP-I&S ^40, 87^	Small molecule	Tc generator	6	n/a	Renal Hepatobiliary	Kidney Spleen	No	(i) SPECT more readily available than PET (ii) Potential use in targeted surgery	(i) Little clinical experience (ii) SPECT lesser resolution and less sensitive than PET
^99m^ Tc-EDDA/HYNIC-iPSMA [41, 88]	Small molecule	Tc generator	6	n/a	Renal	Kidney Salivary gland	No	(i) SPECT more readily available than PET	(i) Little clinical experience (ii) SPECT lesser resolution and less sensitive than PET

**Table 3:** Summary of PSMA-targeted imaging in gliomas.

Authors	Year	*n*	Tumor type	PSMA tracer	Main results
Schwenck et al.	2015	1	GBM	^68^Ga-PSMA	(i) ^68^Ga-PSMA uptake corresponded to contrast enhancement on MRI (ii) No ^68^Ga-PSMA uptake in unaffected brain regions
Unterrainer et al.	2017	1	Gliosarcoma	^68^Ga-PSMA	(i) High ^68^Ga-PSMA uptake, with median SUV_max_ 3.43 (range 2.22-5.27) (ii) Median TBR_max_ 48.93 (range 31.71-75.29)
Sasikumar et al.	2017	6^∗^	GBM	^68^Ga-PSMA	(i) 4/4 GBMs with confirmed recurrence showed both ^68^Ga-PSMA and ^18^F-FDG uptake (ii) Higher TBR with ^68^Ga-PSMA (12.9) than ^18^F-FDG (0.96) in recurrent GBMs (iii) In the newly diagnosed GBM, higher TBR for ^68^Ga-PSMA (22.3) than ^18^F-FDG (1.11) ^∗^These patients were reported again in the Sasikumar (2018) paper
Sasikumar et al.	2018	15^∗^	Glioma	^68^Ga-PSMA	(i) 9/9 GBMs with confirmed recurrence showed ^68^Ga-PSMA uptake (ii) TBR in recurrent GBM ranged 4.07-29.4, versus 1.15 in patient without recurrence (iii) Increased ^68^Ga-PSMA uptake in newly diagnosed and postsurgical GBM (iv) No ^68^Ga-PSMA uptake in postsurgical grade III oligodendroglioma (v) ^68^Ga-PSMA uptake does not correlate to glioma grade: 34.78 in grade II, 11.9-27.0 in grade III, 4.07-134.8 in grade IV
Kunikowska et al.	2018	1	GBM	^68^Ga-PSMA	(i) High ^68^Ga-PSMA uptake with SUV_max_ 23.7
Malik et al.	2018	1	Oligodendroglioma	^68^Ga-PSMA	(i) Better lesion delineation with ^68^Ga-PSMA uptake than ^18^F-FDG
Verma et al.	2019	10	Glioma	^68^Ga-PSMA	(i) Higher SUV_max_ in GBMs (16.93 ± 5.4) than grade II gliomas (2.93 ± 0.3) (ii) Higher TBR in GBMs (13.95) than grade II gliomas (3.42)
Gupta et al.	2020a	1	Recurrent GBM	^68^Ga-PSMA	(i) ^68^Ga-PSMA uptake in postoperative cavity
Gupta et al.	2020b	1	GBM with pseudoprogression	^68^Ga-PSMA	(i) Increased ^68^Ga-PSMA uptake in this “false positive” (ii) SUV_max_ 2.71 (versus 0.52 in normal brain tissue) and TBR 5.21
Kumar et al.	2020	1	Recurrent GBM	^68^Ga-PSMA	(i) ^68^Ga-PSMA uptake in MRI-confirmed recurrent lesion (ii) ^68^Ga-PSMA uptake decreased with regression of lesion posttherapy
Moreau et al.	2020	1	GBM with pseudoprogression	^68^Ga-PSMA	(i) Increased ^68^Ga-PSMA uptake in this “false positive” (ii) SUV_max_ 3.2
Pernthaler et al.	2021	1	Oligodendroglioma	^68^Ga-PSMA	(i) Homogenous high ^68^Ga-PSMA uptake and 18F-fluciclovie uptake (ii) Higher SUV_max_ with ^68^Ga-PSMA (9.7) than 18F-fluciclovine (6.5)
Pilati et al.	2020	1	GBM	^68^Ga-PSMA	(i) High ^68^Ga-PSMA uptake
Zhang et al.	2021	1	Glioma	^68^Ga-PSMA	(i) Heterogeneous ^68^Ga-PSMA uptake, lower in the core of the lesion (grade II tissue) and higher in circumferential foci (grade III tissue)
Kunikowska et al.	2020	15	Recurrent GBM	^68^Ga-PSMA	(i) 15/15 showed increased ^68^Ga-PSMA uptake, which correlated with MRI lesion (ii) Median SUV_max_ 6.5 (range 2.1-14.3), SUV_mean_ 3.5 (1.3-6.1), TBR 96.7 (range 32.2-357.5)
Akgun et al.	2020	35	Glioma	^68^Ga-PSMA	(i) Moderate correlation between tumor grade and SUV_max_ (*r* = 0.53), SUV_mean_ (*r* = 0.55), SUV_peak_ (*r* = 0.50) (ii) Grade II/III gliomas had significantly lower SUV_max_ than GBMs, with a cutoff of 2.3 (iii) LGG versus HGG cutoff for SUV_max_ was 1.15 (iv) ^68^Ga-PSMA was more sensitive (*p* < 0.0%) than MRI, but not more specific (v) Ki-67, mitosis, endothelial proliferation, and necrosis were correlated with SUV values, but ATRX mutation was not
Liu et al.	2021	30	Glioma	^68^Ga-PSMA	(i) PSMA PET had higher SUV_max_ (0.96) and SUV_mean_ (0.94) than FDG PET (0.79, 0.74) (ii) ^68^Ga-PSMA PET was more effective for differentiating HGG from LGG
Salas Fragomeni et al.	2017	3	High-grade glioma	^18^F-DCFPyL	(i) ^18^F-DCFPyL uptake in 3/3 HGGs, with SUV_max_ ranged 5.8-13.5 (ii) No uptake in normal brain tissue
Matsuda et al.	2018	2	High-grade glioma	^89^Zr-Df-IAB2M	(i) In 1, high ^89^Zr-Df-IAB2M uptake in contrast-enhancing MRI lesion (ii) In 1, heterogeneous ^89^Zr-Df-IAB2M uptake with different distribution that 11C-MET
Marafi et al.	2020	1	Recurrent glioblastoma	^18^F-PSMA	(i) Increased uptake of both ^18^F-PSMA and ^18^F-FDG in the MRI lesion (ii) Better differentiation with ^18^F-PSMA than ^18^F-FDG

**Table 4:** Summary of PSMA-targeted imaging in prostate cancer brain metastases.

Authors	Year	*n*	PSMA tracer	Main results
Chakraborty et al.	2015	1	^68^Ga-PSMA	(i) Asymptomatic, avid ^68^Ga-PSMA uptake in 3 separate brain metastases, confirmed by MRI
Dureja et al.	2017	1	^68^Ga-PSMA	(i) Asymptomatic, ^68^Ga-PSMA uptake in 4 brain metastases (SUV_max_ 4.0), confirmed by MRI
Langsteger et al.	2017	1	^68^Ga-PSMA	(i) Asymptomatic, ^68^Ga-PSMA uptake in T4-T10 spinal cord metastases, confirmed by MRI
Chan et al.	2017	1	^68^Ga-PSMA	(i) Symptomatic, ^68^Ga-PSMA uptake in single cerebellar metastasis (SUV_max_ 5.9), confirmed by pathology
Ross et al.	2020	1	^68^Ga-PSMA	(i) Asymptomatic, single brain metastasis detected with ^68^Ga-PSMA but not with standard imaging, confirmed by pathology
Yin et al.	2019	1	^68^Ga-PSMA	(i) Asymptomatic, ^68^Ga-PSMA uptake in multiple brain metastases (SUV_max_ 3-11) and not all detected on MRI, confirmed by pathology
Wei et al.	2017	2	^68^Ga-PSMA	(i) Distinct ^68^Ga-PSMA uptake in cerebral metastases (ii) Decreased uptake as lesion regressed posttherapy
Sathekge et al.	2019	1	^68^Ga-PSMA	(i) Several cerebral metastases avidly showed ^68^Ga-PSMA uptake (ii) Size and ^68^Ga-PSMA uptake decreased posttherapy
McBean et al.	2021	8	^68^Ga-PSMA	(i) 3/8 presented with neurological symptoms (ii) 4/8 had previously undergone ^177^Lu-PSMA therapy (iii) PSMA avidity ranged from not avid to intense, and SUV_max_ ranged 2-21
Parihar et al.	2021	1	^68^Ga-PSMA	(i) ^68^Ga-PSMA uptake in new brain metastases, after ^177^Lu-PSMA and ^225^Ac-PSMA therapy

**Table 5:** Summary of PSMA-targeted imaging in nonprostate cancer brain metastases.

Authors	Year	*n*	Primary lesion	PSMA tracer	Main results
Medina-Ornelas et al.	2017	1	Breast	^68^Ga-PSMA	(i) Symptomatic, brain metastasis showed ^68^Ga-PSMA uptake, confirmed by pathology
Malik et al.	2018	1	Breast	^68^Ga-PSMA	(i) Cerebella metastasis avidly showed ^68^Ga-PSMA uptake but not FDG
Marafi et al.	2020	1	Breast	^68^Ga-PSMA	(i) Intense ^68^Ga-PSMA uptake in cerebral and cerebella metastases, confirmed by MRI (ii) No uptake in normal brain tissue
Arslan et al.	2021	1	Breast	^68^Ga-PSMA	(i) Avid ^68^Ga-PSMA uptake but low FDG uptake in recurrent brain metastasis, confirmed by pathology
Rowe et al.	2015	1	Kidney	^18^F-DCFPyL	(i) Intense ^18^F-DCFPyL uptake in brain metastasis (SUV_max_ 3.9), confirmed by MRI
Yin et al.	2019	1	Kidney	^18^F-DCFPyL	(i) ^18^F-DCFPyL uptake in 3 brain metastases (SUV_max_ 0.5-3.4), confirmed by imaging
Hod et al.	2017	1	Melanoma	^68^Ga-PSMA	(i) Unexpected avid ^68^Ga-PSMA uptake in brain metastasis, confirmed by pathology
Matsuda et al.	2018	1	Lung	^89^Zr-Df-IAB2M	(i) Avid ^89^Zr-Df-IAB2M uptake corresponding to MRI lesion, confirmed by pathology (ii) No uptake in normal brain tissue

**Table 6:** Summary of PSMA-targeted imaging in meningiomas.

Authors	Year	*n*	PSMA tracer	Main results
Bilgin et al.	2016	1	^68^Ga-PSMA	(i) High ^68^Ga-PSMA uptake in brain lesion (SUV_max_ 3.1), diagnosed as meningioma on MRI
Jain et al.	2017	1	^68^Ga-PSMA	(i) Avid ^68^Ga-PSMA uptake in meningioma (SUV_max_ 1.9) but no FDG avidity
Sasikumar et al.	2017	1	^68^Ga-PSMA	(i) Stronger ^68^Ga-PSMA uptake (SUV_max_ 14.6, TBR 29.2) than FDG (SUV_max_ 6.44, TBR 0.74) (ii) Meningioma diagnosis confirmed by pathology
Gupta et al.	2020	1	^68^Ga-PSMA	(i) Avid ^68^Ga-PSMA uptake, diagnosis confirmed by MRI
Courtney et al.	2021	1	^68^Ga-PSMA	(i) ^68^Ga-PSMA uptake demonstrated in a meningioma
Junqueira et al.	2021	1	^68^Ga-PSMA	(i) Strong ^68^Ga-PSMA uptake demonstrated in an intraventricular meningioma (SUV_max_ 12.1)
Calabria et al.	2017	1	^64^Cu-PSMA	(i) ^64^Cu-PSMA uptake in MRI-confirmed meningioma was SUV_max_ 3.8 at 1 h postinjection, 3.9 at 4 h postinjection
Haemels et al.	2020	1	^18^F-PSMA	(i) Moderately avid ^18^F-PSMA uptake in pathology-confirmed meningioma

## Data Availability

The data supporting this systematic review article are from previously reported studies included within the article and which have been cited.

## Abbreviations

AUC: Area under the ROC curve
BBB: Blood-brain barrier
CNS: Central nervous system
CT: Computerized tomography
^18^F-DCFPyL: 2-(3-(1-Carboxy-5-(6-[^18^F]fluoro-pyridine-3-carbonyl)-amino-pentyl)-ureido)-pentanedioic acid
FDG: Fluorodeoxyglucose
GBM: Glioblastoma
GCPII: Glutamate carboxypeptidase II
HGG: High-grade glioma
IHC: Immunohistochemistry
LGG: Low-grade glioma
mAb: Monoclonal antibody
MRgFUS: MRI-guided focused ultrasound
MRI: Magnetic resonance imaging
NAAG: N-Acetyl-L-aspartyl-L-glutamate
PCNSL: Primary central nervous system lymphoma
PET: Positron emission tomography
PMPA: PSMA-receptor inhibitor 2-(phosphonomethyl)pentane-1,5-dioic acid
PSMA: Prostate-specific membrane antigen
SPECT: Single-photon emission computerized tomography
SUV: Standardized uptake value
TBR: Tumor to background ratio
VWF: von Willebrand factor
^89^Zr-Df-IAB2M: 89-Zirconium-desferrioxamine anti-PSMA minibody.
